# Improvement of Sinapine Extraction from Mustard Seed Meal by Application of Emerging Technologies

**DOI:** 10.3390/foods12030520

**Published:** 2023-01-23

**Authors:** Morad Chadni, Nadia Boussetta, Cédric Guerin, Fabien Lagalle, Aya Zoghlami, Patrick Perré, Florent Allais, Nabil Grimi, Irina Ioannou

**Affiliations:** 1 URD Agro-Biotechnologie Industrielles, CEBB, AgroParisTech, FR CNRS 3417 Condorcet, 51110 Pomacle, France; 2 Université de Technologie de Compiègne, ESCOM, TIMR (Integrated Transformations of Renewable Matter), Centre de Recherche Royallieu, FR CNRS 3417 Condorcet, CEDEX CS 60 319, 60203 Compiègne, France; 3 CentraleSupélec, Laboratoire de Génie des Procédés et Matériaux, Centre Européen de Biotechnologie et de Bioéconomie (CEBB), Université Paris-Saclay, 3 rue des Rouges Terres, 51110 Pomacle, France

**Keywords:** high-voltage electrical discharge, ultrasound, supercritical CO_2_, sinapine extraction, mustard seed meal, phenolic compounds, *Brassica juncea*

## Abstract

Sinapine is a phenolic compound found in mustard (*Brassica juncea*) seed meal. It has numerous beneficial properties such as antitumor, neuroprotective, antioxidant, and hepatoprotective effects, making its extraction relevant. In this study, the extraction of sinapine was investigated using three methods: (i) from a mustard seed meal defatted by a supercritical CO_2_ (SC-CO_2_) pretreatment, (ii) by the implementation of high-voltage electrical discharges (HVEDs), (iii) and by the use of ultrasound. The use of SC-CO_2_ pretreatment resulted in a dual effect on the valorization of mustard seed meal, acting as a green solvent for oil recovery and increasing the yield of extracted sinapine by 24.4% compared to the control. The combination of ultrasound and SC-CO_2_ pretreatment further increased the yield of sinapine by 32%. The optimal conditions for ultrasound-assisted extraction, determined through a response surface methodology, are a temperature of 75 °C, 70% ethanol, and 100% ultrasound amplitude, resulting in a sinapine yield of 6.90 ± 0.03 mg/g dry matter. In contrast, the application of HVEDs in the extraction process was not optimized, as it led to the degradation of sinapine even at low-energy inputs.

## 1. Introduction

Mustard (*Brassica juncea)* seed meal is a by-product generated during mustard production. It represents 60% (*w*/*w*) of the processed seeds’ by-products [1,2]. This by-product is mainly undervalued in methanization [3]. However, it contains many primary metabolites such as lipids, proteins and carbohydrates, but also secondary metabolites such as glucosinolates, phytates and phenolic compounds [4,5]. The phenolic compounds present in mustard seed meal are mainly represented by condensed tannins, flavonoids and phenolic acids [6,7,8]. They represent between 0.78% and 1% of the dry matter of the mustard seed meal [9].

Sinapine, a choline ester of sinapic acid, is the main phenolic compound in mustard seed meal, comprising more than 80% (*w*/*w*) of the total phenolic compounds, and has been found to exhibit antioxidant, antibacterial, and UV-filtering properties [10,11]. Additionally, sinapic acid has been shown to possess antioxidant, antimicrobial, anti-inflammatory, anticancer, and anxiolytic activities, making the recovery of sinapine and/or sinapic acid from mustard by-products a potentially interesting area of research [12].

There is a limited number of studies dedicated to the extraction of sinapine from mustard by-products in comparison to the number of studies conducted on other Brassica species, such as rapeseed (*Brassica napus*). Conventional solvent extraction utilizing solvents such as methanol, ethanol and their water mixtures are most commonly used [9,13]. However, there is a need for methods that are more environmentally friendly and more efficient. Alternative techniques, such as ultrasound [14,15], microwaves [16], enzyme-assisted extraction [17], pressurized liquids [18], supercritical fluids [19], and high-voltage electrical discharges [20], are being increasingly studied as unconventional and innovative approaches for biomolecule extraction.

In this paper, three extraction technologies were implemented to improve the extraction of sinapine from mustard seed meal: high-voltage electrical discharges (HVEDs), supercritical CO_2_ and ultrasound (US). HVEDs have already shown their ability to improve the extraction yields of high-added-value compounds by increasing the cellular permeability [21,22,23,24,25]. This technique delivers energy directly into an aqueous solution through a plasma channel formed by a high-current/high-voltage electrical discharge between two immersed electrodes. Large ranges of current (10^3^–10^4^ A), voltage (10^3^–10^4^ V) and frequency (10^−1^–10 Hz) are used. HVEDs require short times of treatment (a few milliseconds) and low energy consumption (10–100 kJ/kg) [26]. To our knowledge, the use of high-voltage electrical discharges has only been carried out on mustard by-products for the extraction of glucosinolates [27]. Indeed, the use of high-voltage electrical discharges improved the extraction yield of sinapine from rapeseed [28]. 

The use of supercritical CO_2_ for the extraction of sinapine from mustard seed meal typically involves a pretreatment step of defatting the mustard seed meal. While supercritical CO_2_ has been previously utilized for extracting oil from mustard seeds, the effect of this defatting step on the extraction of sinapine specifically has not been well studied [29]. 

Ultrasound-assisted extraction (UAE) relies on ultrasound energy and the nature of the solvent to recover specific compounds from various biomasses. UAE offers several advantages including reduced time and energy requirements, low-temperature extraction, and preserved extract quality. This technique extracts bioactive molecules from plant matrices through the use of high-intensity acoustic waves. These cause disruptions in the plant tissue due to the physical forces developed during acoustic cavitation. The destabilization of cell walls contributes to the release of soluble components into the solvent in minimal time by improving their mass transport [30].

The quantification of phenolic compounds extracted from Brassica plants is often carried out by the Folin–Ciocalteu method, which makes it difficult to accurately assess the improvement in sinapine yield or process efficiency [15,31,32,33].

The main objective of this paper was to investigate the effect of three extraction technologies (HVEDs, US and SC-CO_2_) on the sinapine extraction yield. In this study, SC-CO_2_ pretreatment was applied to extract oil from mustard seed meal; therefore, it was considered as a preliminary treatment for sinapine extraction. Ultrasound and a high-voltage electrical discharge were applied as physical and electrical treatments to improve the extraction of sinapine. Concerning the ultrasound-assisted extraction, an optimization by response surface methodology (RSM) was carried out to obtain the optimal treatment conditions and the extraction kinetics were modeled. Observations of the treated matter by environmental electron scanning microscope (ESEM) were finally proposed to qualitatively describe the effect of the extraction technologies at the microscopic scale.

## 2. Materials and Methods

### 2.1. Chemicals and Materials

Ethanol (purity > 99.9%), methanol (purity > 99.9%), acetonitrile (purity > 99.9%) and formic acid (purity > 98%) were purchased from VWR, France.

The biomass used was mustard (*Brassica juncea*) seed meal. It is made up of seed hulls, seed fragments and residual cotyledons. It was generated as a by-product of the cold mechanical pressing of dry mustard seeds and was kindly obtained from the company Charbonneaux-Brabant (Reims, France) and stored in the dark at 4 °C.

### 2.2. Conventional Extraction of the Sinapine 

The conventional extraction was carried out in a 500 mL three-necked flask with a condensation column at 75 °C with 70% (*v*/*v*) ethanol as the solvent for 30 min. These conditions were chosen based on our previous works [1,13]. After centrifugation at 4 °C and 4713 g for 10 min, the liquid extract was isolated from the mustard seed meal. It was assumed that the ethanol/water mixture was homogeneous and that the concentration of sinapine was uniform throughout the liquid phase.

### 2.3. Extraction Technologies 

#### 2.3.1. High-Voltage Electrical Discharges (HVEDs)

Mustard seed meal was treated with HVEDs using a 40 kV–10 kA pulsed high-voltage generator (Basis, Saint Quentin, France) and a 1 L cylindrical batch-treatment chamber with needle-plate electrodes. The stainless-steel needles had a diameter of 10 mm and the grounded disc electrodes had a diameter of 35 mm, with a distance of 10 mm between the electrodes. The treatment utilized low-inductance condensers charged by the high-voltage supply to generate electrical discharges through an electrical breakdown in the liquid extraction medium. The HVED generator provided exponentially shaped pulses with a pulse repetition rate of 0.5 Hz.

The HVED-treatment chamber was initially filled with 20 g of mustard seed meal, which was mixed with distilled water at a liquid-to-solid ratio of 15 (*w*/*w*). The number of electrical pulses applied ranged from 50 to 400, corresponding to an energy consumption range of 20 to 160 kJ/kg of suspension. 

#### 2.3.2. Supercritical CO_2_

The use of supercritical CO_2_ can be considered as a pretreatment but also allows the extraction of lipids from the mustard seed meal. The equipment used was a supercritical fluid extraction system (SFE PROCESS, Nancy, France), supplied with a 1 L extraction tank and two 0.2 L separators in series. Carbon dioxide (99.99% purity) was obtained from Linde PLC (Lyon, France) and used as the extraction solvent. First, 150 g of mustard seed meal were placed in a 1 L sample cartridge. The CO_2_ flow (downward) was kept constant at 60 g/min during the experiments. Based on the literature data [28,34,35,36], the pressure was kept at 350 bar and the temperature at 40 °C. The oil obtained was weighed and analyzed to determine the profile of the lipids extracted. The defatted mustard seed meal then underwent an extraction according to the protocol described in part 2.2. The sinapine content was measured by UHPLC.

#### 2.3.3. Ultrasound-Assisted Extraction 

Ultrasounds were applied simultaneously with the extraction of the sinapine. The setup used was a 2 L stirred glass reactor equipped with an ultrasonic generator (400 W-24 KHz) and a titanium sonotrode (Reference S24d22D) with a diameter of 22 mm (Hielscher Ultrasonics, Germany). The extraction temperature was maintained constant by circulating glycerol in an outer jacket connected to a cryothermostat. The stirring speed was set at 250 rpm in all experiments, and the solvent volume was 600 mL. Extractions were performed with ultrasound in continuous mode at different amplitudes (0–100%).

A Box–Behnken experimental design with response surface methodology (RSM) was selected to optimize the ultrasound-assisted extraction of sinapine. The design was created by the MODDE software (Sartorius, Sweden) and included 15 experiments, with a triplicate at the central point. The independent variables were the ethanol percentages in water (0, 35, and 70%), the extraction temperature (25, 50, and 75 °C) and the ultrasound amplitude (0, 50, and 100%), while the solid-to-liquid ratio and the extraction time were fixed at 1, 20 and 30 min, respectively. Experiments were run randomly to minimize the impact of unexplained variability, and a second-order polynomial equation was used to adjust the experimental data (Equation (1)).

(1) $$Y=\beta_{0}+\sum_{i=1}^{k}\beta_{i}X_{i\;}+\sum_{i=1}^{k}\beta_{ii}X_{ii\;}+\sum_{i=1}^{k}\sum_{j=i+1}^{k}\beta_{ij}X_{ij\;}+\varepsilon$$

where Y represents the response; $\beta_{0}$ is the model intercept;$\;\beta_{i}$, $\beta_{ii}$ and $\beta_{ij}$ are the coefficients of the linear, quadratic, and interactive effects, respectively; $X_{i\;}$, and $X_{j}$ represent the coded independent factors; k equals the sum of variables tested (k = 3 in this work) and ɛ is the discrepancy between the predicted and experimental values.

Analysis of variance was conducted at a 95% confidence level to evaluate the accuracy and relevance of the model. The model’s coefficients were considered statistically significant if the *p*-value from the Student’s *t*-test was less than 0.05.

### 2.4. Kinetic Modeling of the Extraction of Sinapine 

The extraction kinetics of sinapine were investigated at the best conditions, obtained with ultrasound, and compared to the nontreated control and SC-CO_2_-defatted sample. 

The extraction rate and kinetic parameters of Peleg’s model (Equation (2)) were used to investigate the yield of the sinapine extraction under different conditions. The experimental data for the extraction of polyphenols were best described by the Peleg model [37,38].

(2) $$Y\;\left(t\right)=\frac{t}{K_{1}+K_{2}.t\;}$$

Here, Y (t) represents the extraction yield of sinapine at time t ; $K_{1}$ and $\;K_{2}$ represent Peleg’s rate constant (min. g dry matter (DM) /mg) and Peleg’s capacity constant (g DM/mg), respectively.

$K_{1}$ refers to the extraction rate $R_{0}$ (mg/g DM.min) at the very beginning (t = $t_{0}$):

(3) $$R_{0}=\frac{1}{K_{1}}$$

$\;K_{2}$ refers to the maximum extraction yield, i.e., equilibrium yield of extracted sinapine ($Y_{e}$) when t → ∞. The relationship between the equilibrium yield and $\;K_{2}$ is shown in Equation (4):

(4) $$Y\;{\left(t\right)}_{t\to \infty }=Y_{e}=\frac{1}{K_{2}}$$

The kinetic parameters of Peleg’s model were determined using Excel software. The goodness of fit between the experimental yields and the model-predicted yields was quantified using the determination coefficient (R^2^) and root-mean-square error (RMSE) as shown in (Equation (5)).

(5) $$RMSE=\sqrt{\frac{\sum_{i=1}^{n}{\left(y_{exp}-y_{pred}\right)}^{2}}{n}}$$

The variable  n represents the number of experimental data points included in a kinetic plot. $y_{exp}$ and $y_{pred}$ refer to the experimental and model values, respectively, at the i-th point.

### 2.5. Analytical Measurements

#### 2.5.1. Quantification of Sinapine by UHPLC

The extracts obtained from mustard seed meal were filtered through a 0.20 μm filter and then analyzed using reversed-phase ultra-high-performance liquid chromatography with diode array detection (UHPLC-DAD) on an Ultimate 3000 system (Dionex, ThermoFisher, Sunnyvale, CA, USA). A Thermo Scientific C18 Accucore aQ column (100 × 3 mm, 2.6 μm particle size) was used with a gradient elution of water (solvent A), acetonitrile (solvent B), and 0.1% formic acid (solvent C). The starting solvent composition was 45% A, 5% B, and 50% C, and the gradient of solvent B was 5% at 0 min, 10% at 0.990 min, 15% at 3.190 min, 30% at 7.440 min, and 5% at 8.510 min, while solvent C remained constant. The column was maintained at 48 °C and the flow rate was 0.8 mL/min. The total run time was 13 min with a 20 μL injection volume, and the wavelength of detection was 320 nm. Sinapine standards were obtained from Sigma-Aldrich (Saint-Quentin-Fallavier, France).

#### 2.5.2. Gas Chromatography–Tandem Mass Spectrometry (GC–MS/MS)

Oil samples were derivatized before GC analysis following the protocol proposed in the literature [39]. In short, samples were filtered using a 0.22 µm filter in PTFE, diluted 50-fold in chloroform. Next, 40 µL were transferred to a vial, 40 µL of a tridecanoic acid solution at 12 mg/L, 320 µL of chloroform and 200 µL of trimethylsulphonium hydroxide (TMSH) were added. The vial was then stirred for one minute and was left standing still for one hour. The derived oil was then diluted 100-fold before injection into the GC–MS/MS. The efficiency of the derivatization was assessed using tridecanoic as an internal standard.

The quantification of fatty acid methyl esters (FAME) was performed using a GC-2010 Plus gas chromatograph coupled with a GCMS-TQ8040 triple quadrupole mass spectrometer (Shimadzu, Kyoto, Japan). The separation was performed on an HP-88 capillary column (Agilent) with dimensions of 100 m × 0.25 mm × 0.20 µm using helium (99.9999%) as the carrier gas at a flow rate of 2 mL/min. The injection volume and temperature were 1 µL and 240 °C, respectively, and the samples were injected in splitless mode. The oven temperature was initially set at 50 °C for 1 min and then increased to 180 °C at a rate of 20 °C/min and held for 5 min. It was subsequently increased at rates of 10 °C/min to 200, 210, 220, and 230 °C for 5, 5, 1, and 6 min, respectively.

The total run time was 34.5 minutes. Detection was carried out using the triple quadrupole in electron ionization (EI) mode with an energy of 70 eV, and the temperature of the ion source and interface were set at 200 °C and 210 °C, respectively. The acquisition was performed in multiple-reaction mode (MRM).

The compounds were identified by comparing their retention times with standards. For each compound, three different transitions were recorded. One transition was used to quantify the compound using the area of the chromatographic peak, while the two other transitions were used as qualifiers. The intensity ratio between the quantifiers and qualifiers was also used for additional identification. C13:0 was used as the internal standard for calibration. The FAME standard was the Supelco 37 component FAME mix (CRM47885) from Sigma-Aldrich (Saint-Quentin-Fallavier, France).

To visualize any unquantified compounds, all samples were also scanned using the third quadrupole from 50 to 400 *m*/*z*.

#### 2.5.3. Environmental Scanning Electron Microscopy (ESEM)

An environmental scanning electron microscope (ESEM, Quanta 200, FEI Company, Gothenburg, Sweden) was used to obtain microscopic images of the samples. The images were captured in low-vac mode at a voltage of 10 kV, a pressure of 133 Pa, and a beam spot size of 4.5, with a working distance of approximately 6 mm. The low-vac mode allows samples to be imaged without any coating even though they are not electrically conductive. The tension was reduced to limit the beam penetration and the working distance was a compromise between the detector signal and the degradation of the electron beam in the chamber, which was not in secondary vacuum.

The samples were placed on a carbon conductive double-sided adhesive disc without any further preparation.

### 2.6. Statistical Analysis

The standard deviation was calculated from three repetitions of the central point in the design of the experiment. The kinetic study was conducted twice, and the average sinapine content was determined and accompanied by its standard deviation. The Tukey test was performed to detect significant distinctions among the groups using R Software [40].

## 3. Results and Discussions

### 3.1. Effect of the HVEDs on the Sinapine Extraction

**Figure 1:**
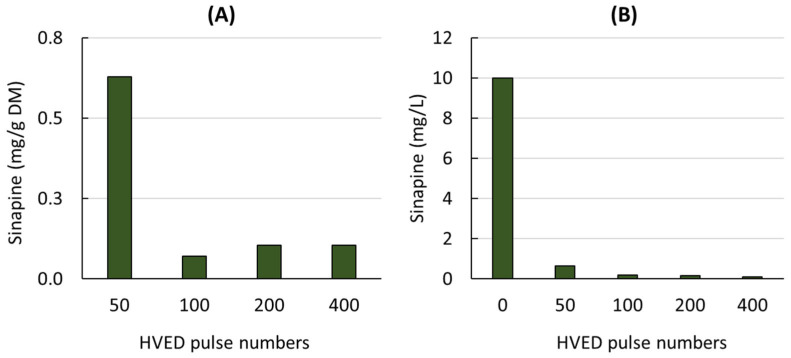
Sinapine content of liquid extracts obtained from mustard seed meal (**A**) and from model solution containing sinapine (**B**) treated by different HVED pulse numbers.

The sinapine content of the liquid extracts obtained from mustard seed meal treated with different numbers of HVED pulses is shown in Figure 1A.

As the number of pulses increased, the sinapine content decreased rapidly to very low values (0.1 mg/g DM). In contrast to the results reported for other raw materials and other types of phenolic compounds [20,23,24,41], HVEDs had a negative effect on the sinapine content, suggesting that HVEDs may cause the degradation of this component. 

To check the effect of HVEDs on the sinapine stability, HVEDs were also applied to a model solution containing sinapine (10 mg/L) (Figure 1B). A similar behavior was observed for the mustard seed meal extracts and the model sinapine solution. After 50 pulses, very little sinapine was detected. Sinapine degradation may be related to the production of active species during HVED. This negative effect of HVED has also been previously reported on catechin, epicatechin, quercetin-3-*O*-glucoside and kaempferol-3-O-glucoside from grape pomace [42].

In 1991, Bogomaz et al. described the effects of HVEDs in water as comprising two main mechanisms: (i) local action due to the chemical reactions in the vicinity of the plasma and (ii) nonlocal action due to a powerful shock wave (10–20 MPa) and UV radiation (200 to 400 nm) [43]. The shock waves produced create a highly turbulent mixing environment and mechanically rupture cell membranes, which enhances sinapine extraction. However, HVEDs can also generate hydroxyl radicals through water photodissociation and produce atomic hydrogen and ozone, which may damage the extracted sinapine through oxidative chemical reactions. When a high number of discharges is used, the quantity of oxidizing species produced increases and may oxidize sinapine. In 2004, Chen et al. observed the degradation of phenols by electrical discharge [44]. The degradation of phenols by electrical discharge is thought to result from the reaction of the hydroxyl radical (•OH) with phenols, leading to the formation of phenoxyl radicals. Phenoxyl radicals may also be directly formed through the effect of the ultraviolet light produced by the discharge. Sinapine degradation may also be caused by the formation of ozone during HVED, which reacts with water molecules to produce hydrogen peroxide, which then decomposes to form hydroxyl radicals. 

Previous studies on certain biomasses, such as grape pomace, have shown that the application of HVEDs can initially increase the total phenol content before decreasing it [42]. Similarly, the sinapine content in mustard seed meal was found to immediately decrease after the first HVED pulses, suggesting that sinapine may have a greater sensitivity to the oxidized species produced by HVED, making it an unsuitable method for the extraction of sinapine from mustard seed meal.

### 3.2. Effect of the SC-CO_2_ Extraction as Pretreatment Technology on the Sinapine Extraction

**Figure 2:**
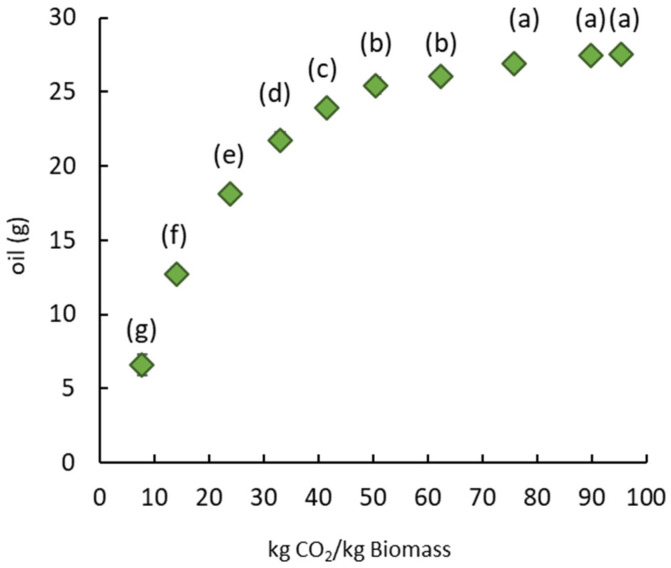
Amount of oil from mustard seed meal as a function of the mass of CO_2_ at 350 bar and 40 °C. Significant differences (*p* > 0.05) between bars, as determined by the Tukey test at a 95% confidence level, are indicated by distinct labels.

The application of SC-CO_2_ leads to oil recovery and pretreatment of the mustard seed meal to improve the extraction of sinapine. The SC-CO_2_ extraction parameters were chosen based on the literature [34,35,36]. The defatted solid residue remaining after SC-CO_2_ extraction was used for the sinapine extraction. The quantity of oil obtained is plotted in Figure 2 as a function of the quantity of CO_2_ used.

The quantity of oil extracted increased with the quantity of CO_2_ used per kg of dry mustard seed meal and then stabilized at around 75 kg CO_2_ for 1 kg of dry mustard seed meal. This ratio corresponded to an extraction time of 2 hours and produced 19 g of oil per 100 g of dry matter. The mustard seed meal residue after SC-CO_2_ extraction was dry and discolored, suggesting that almost all the neutral lipids were extracted. The extracted oil was characterized, and the fatty acid composition was detailed in order to qualify the oil obtained. Thereby, Table 1 presents the percentage of saturated, monounsaturated and polyunsaturated fatty acids, as well as the ratios of saturated/unsaturated, Omega 3/Omega 6, saturated/Omega 6 and saturated/Omega 3 fatty acids.

**Figure 3:**
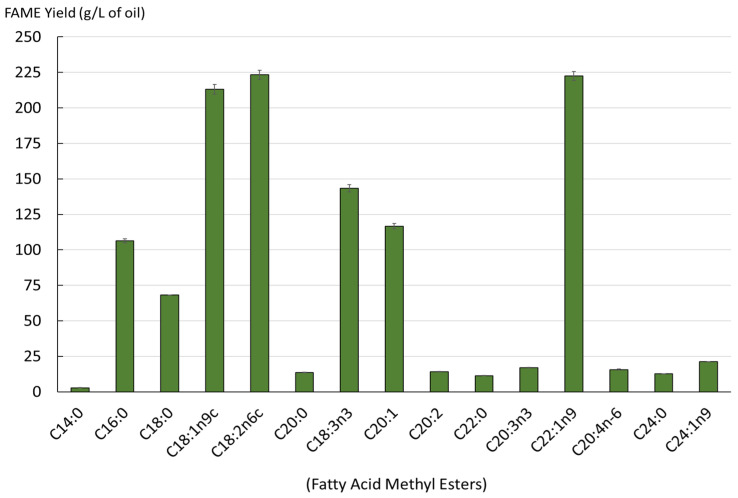
Yield of fatty acids in the extracted lipids from SC-CO_2_ (mg/L, as FAMEs).

Monounsaturated fatty acids were the main components, accounting for 47% of the total fatty acids found in mustard seed meal. The polyunsaturated fatty acids represented 34.40% of the total fatty acids in the extracts obtained, while the saturated fatty acids were around 17.87%. The fatty acid profile of lipids is shown in Figure 3.

**Figure 4:**
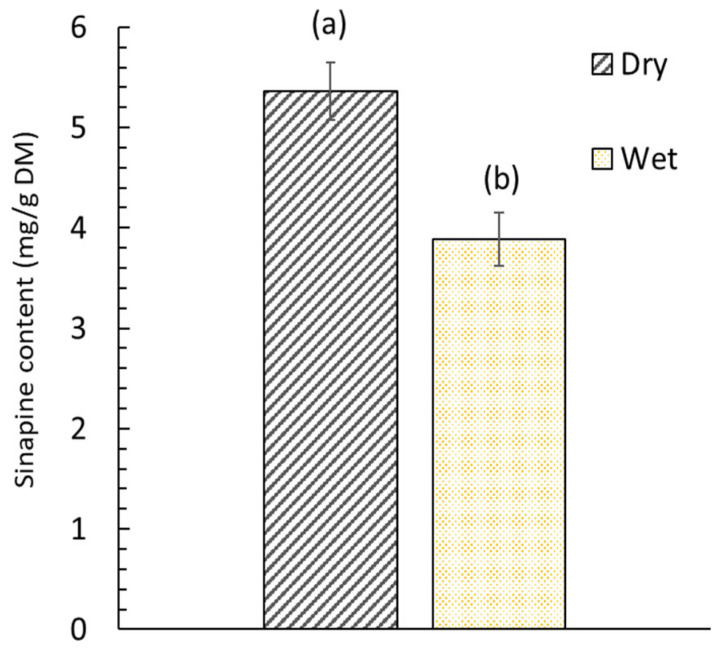
Sinapine content in dry (5.5% *w*/*w* moisture content) and wet (52.4% moisture content) mustard seed meal. Significant differences (*p* > 0.05) between bars, as determined by the Tukey test at a 95% confidence level, are indicated by distinct labels.

The obtained oil contained a diverse range of fatty acids. A total of 15 different acids were identified. The lipids obtained had a high content of erucic acid (C22:1n9), gondoic acid (C20:1), oleic acid (C18:1n9c), alpha-linolenic acid (C18:3n3) and linoleic acid (C18:2n6c). Figure 4 illustrates the impact of the initial step of the SC-CO_2_ extraction of oil from dry and wet mustard seed meal on the subsequent step of sinapine extraction. Sinapine recovery was subsequently performed using the conventional extraction.

The extraction efficiency of sinapine was highly related to the moisture content of the mustard seed meal. The sinapine content of wet mustard seed meal was lower by 27.6% compared to that obtained from dry mustard seed meal. The difference between these two values can be considered significant (*p* < 0.05). This can be attributed to the fact that the sinapine was co-extracted at the same time as the oil during the SC-CO_2_ pretreatment for wet products. Indeed, the water present in wet mustard seed meal plays the role of co-solvent and promotes the extraction of sinapine [45]. From dry mustard seed meal, no sinapine was extracted due to the low solubility of sinapine (polar compound) in nonpolar solvents (SC-CO_2_). The effect of water during the SC-CO_2_ is related to the establishment of hydrogen bonds as well as dipole–dipole or induced dipole interactions between the compound and water, but it also impacts the extraction through solvent–co-solvent interaction [46]. Thus, drying the matter is important to not losing a portion of the sinapine.

### 3.3. Effect of the Ultrasound-Assisted Extraction

The extraction of bioactive molecules from biomass can be greatly influenced by various parameters such as temperature, time, treatment intensity, and solvent composition. To optimize the sinapine extraction yield, a Box–Behnken design was used to study the effects of ultrasound amplitude, extraction temperature, and solvent composition. The results of this optimization are shown in Table 2. 

The coefficients of the model predicting the yield of sinapine and the required parameters for the validation of the model are presented in Table 3.

The goodness of fit of the models to the experimental data was evaluated using the coefficients of determination, R^2^ and R^2^ adj. The obtained R^2^ value of 0.919 and R^2^ adj of 0.887 indicate a strong fit of the model to the data.

According to Table 2, the yield of sinapine varied significantly for each extraction condition from 0 to 6.37 mg/g DM. As shown in Table 3, the three variables studied had a significant effect (*p*-value < 0.05), as did the quadratic term of ethanol. The interaction terms were not significant. 

To predict the yield of sinapine, a second-order polynomial model was applied. Insignificant coefficients were deleted to obtain a reduced model. The elaborated equation in terms of coded factors is shown as follows:

(6) $$Sinapine\;yield\;\left(mg/g_{DM}\;\right)=2.627+1.518\times T+1.146\times E+0.741\times A+0.834\times E^{2}$$

**Figure 5:**
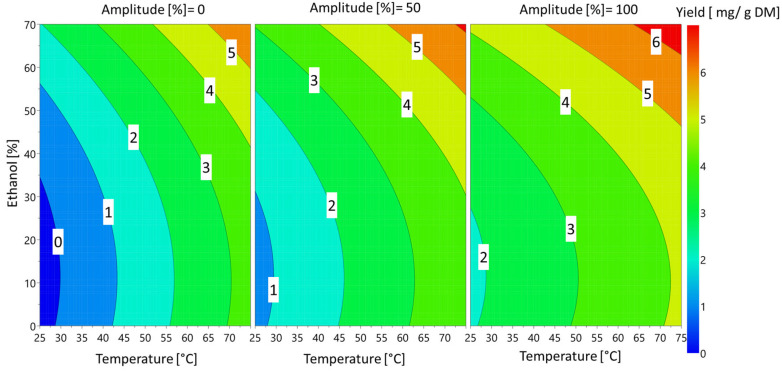
Response 4D contour plot of the effects of ethanol percentage, ultrasound amplitude and temperature on sinapine extraction yield.

These effects can also be visualized Figure 5 on the 4D contour plots.

**Figure 6:**
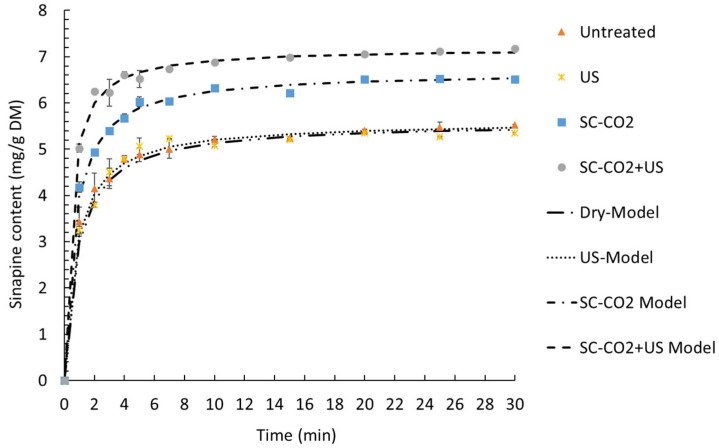
Kinetics of extraction of sinapine at 75 °C and 70% ethanol with different extraction technologies. The extractions were performed for untreated and pretreated samples (ultrasound, SC-CO_2_ and SC-CO_2_ + US).

The temperature and percentage of ethanol had the greatest influence on sinapine yield. These findings are in accordance with previous works [9]. The amplitude of the ultrasound had a slight significant effect on the response. Regardless of the amplitude, the extraction of sinapine was more efficient at high temperatures in the presence of ethanol. The yield of sinapine extraction using water was low, but increasing the temperature slightly improved the release of sinapine in water, resulting in a yield that was approximately 50% of the maximum yield obtained. Based on the predictive models, the optimal conditions to maximize the sinapine yield were 70% ethanol, 75 °C and 100% amplitude, with a value (y_predicted_) of 6.56 mg/g DM. To check the predictive value, the kinetics of the extraction under the best conditions were investigated. The kinetics of sinapine content for untreated and pretreated samples are presented in Figure 6. 

The experimental data were fitted with Peleg’s model, as presented in Figure 6. The kinetic parameters of the model, the coefficients of determination and the RMSE are presented in Table 4.

A good fit of a proposed kinetic model is obtained when R^2^ is close to 1 and the RMSE is near 0. In the present study, the R^2^ ranged from 0.996 to 0.998 and the RMSE ranged from 0.082 to 0.139. Therefore, Peleg’s model describes our experimental data very well.

The extraction rate constant (K_1_) and the extraction extent constant (K_2_) decreased in the following order: without pretreatment > US > SC-CO_2_ > SC-CO_2_ + US. This indicates that the fastest and most efficient extraction was the one combining the SC-CO_2_ pretreatment and ultrasound-assisted extraction. Thus, the highest initial extraction rate (R_0_) was obtained with SC-CO_2_ + US (17.697 ± 0.472 mg/g DM min), followed by SC-CO_2_ (10.517 ± 0.489 mg/g DM min). The same trend was observed for the equilibrium extraction yield (Ye) with a value of 5.565 ± 0.014 mg/g DM without pretreatment, 5.531 ± 0.074 mg/g DM for US, 6.636 ± 0.037 mg/g DM with SC-CO_2_ and 7.194 ± 0.007 mg/g DM by combining the SC-CO_2_ pretreatment and ultrasound-assisted extraction. 

Figure 6 demonstrates that the experimental values were nearly equal to the predicted values (Y_experimental_ = 6.9 ± 0.03 mg/g DM). Moreover, the equilibrium extraction time was achieved in 10 minutes, which allowed for a reduction in the extraction time compared to previous work (Reungoat et al., 2020). Combining the SC-CO_2_ pretreatment and ultrasound-assisted extraction was found to be very effective compared to the control (dry), ultrasound alone and SC-CO_2_ pretreatment alone. Indeed, after 10 min, SC-CO_2_ + US increased the yield by 32% and 8.9% compared to the control (dry and ultrasound alone) and the SC-CO_2_ pretreatment, respectively. 

### 3.4. Environmental Scanning Electron Microscopy (ESEM) Observations

ESEM of the mustard seed meal pretreated by SC-CO_2_ and ultrasound corresponding to the maximum extraction yields were studied. 

**Figure 7:**
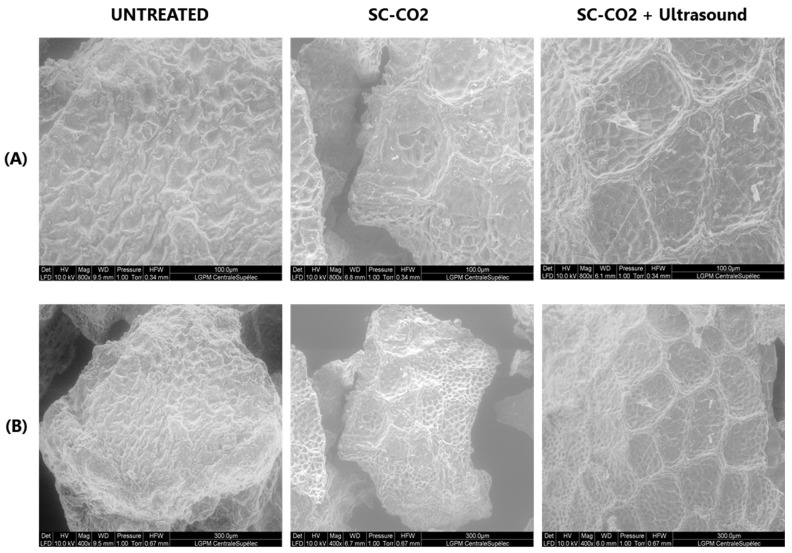
ESEM images of mustard seed meal subjected to SC-CO_2_ and ultrasound: (**A**) 400×–10 KV; (**B**) 800×–10 KV.

Figure 7 shows that the surface of the untreated sample appears to be unaltered, with no cracks or cavities visible. However, the surface morphology of the mustard seed meal after SC-CO_2_ pretreatment exhibits significant variations. The surface exhibits an abnormal porosity due to the diffusion of SC-CO_2_ through the hulls and the removal of the lipid fraction. SC-CO_2_ pretreatment disrupts the mustard cells during oil extraction, making it easier to remove the cell contents. This is reflected in the fact that sinapine extraction from SC-CO_2_-treated mustard seed meal increased by 24.4% compared to untreated mustard seed meal as a result of SC-CO_2_ pretreatment. The SC-CO_2_-pretreated mustard seed meal subjected to ultrasound treatment exhibited surface peeling, erosion, and particle breakdown due to micro-jetting caused by the implosion of cavitation bubbles in the product proximity [47]. The damage to the mustard seed meal induced by ultrasound increased the sinapine yield by 8.9% compared to the sample without ultrasound treatment.

## 4. Conclusions

This work highlights the impact of different extraction technologies on sinapine extraction from mustard seed meal. High-voltage electrical discharges are not suitable for the extraction of sinapine from mustard seed meal. A higher degradation of sinapine was observed after HVED treatment. However, the combination of SC-CO_2_ and ultrasound technologies improved the yield of extraction. SC-CO_2_ pretreatment is not only effective for oil extraction but allows more sinapine to be recovered. The increase obtained with the SC-CO_2_ pretreatment could be attributed to the removal of the lipid fraction and the change in the surface morphology of the mustard seed meal. The application of ultrasound during the extraction of mustard seed meal treated with SC-CO_2_ also improved the yield of sinapine. The application of these technologies generated an increase of 10.13% on the extraction yield. The extraction of the lipid fraction opens the way to an integral valorization of the biomass, making it possible to reduce the cost of the sinapine extraction process.

## Figures and Tables

**Table 1:** Summary of fatty acid composition of mustard oil obtained by SC-CO_2_.

SAT (%)	MONOSAT (%)	POLYSAT (%)	SAT/UNSAT Ratio	Omega 3/ Omega 6 Ratio	SAT/ Omega 6 Ratio	SAT/ Omega 3 Ratio
17.87	47.71	34.40	0.22	0.67	0.90	1.34

SAT, saturated; MONOSAT, monounsaturated; POLYSAT, polyunsaturated; UNSAT, unsaturated (= MONOSAT + POLYSAT).

**Table 2:** Experimental matrix design and response values.

Parameter Values				Response
Run	Temperature (°C)	Ethanol (%)	Amplitude (%)	Sinapine (mg/g DM)
1	25	0	50	0.15
2	75	0	50	3.71
3	25	70	50	2.68
4	75	70	50	6.37
5	25	35	0	0.00
6	75	35	0	3.15
7	25	35	100	2.65
8	75	35	100	4.39
9	50	0	0	2.19
10	50	70	0	4.18
11	50	0	100	3.21
12	50	70	100	5.2
13	50	35	50	3.44
14	50	35	50	2.16
15	50	35	50	2.6

**Table 3:** Coefficients and statistical parameters of the model.

Factors	Centered and Scaled Coefficients	Coefficient Values	*p*
Constant	β0	2.627	0.000 *
Temperature (T)	β1	1.518	0.000 *
Ethanol (E)	β2	1.146	0.000 *
Amplitude (A)	β3	0.741	0.024 *
T × T	β11	−0.326	0.382
E × E	β22	0.834	0.016 *
A × A	β33	0.140	0.697
T × E	β12	0.032	0.924
T × A	β13	−0.352	0.331
E × A	β23	1.167 × 10^−7^	1
Validation parameters for reduced model			
R^2^		0.919	
R^2^ adj		0.887	

* Significant parameters (*p* < 0.05).

**Table 4:** Kinetic and validation parameters for Peleg’s model describing the extraction yield of sinapine.

	K_1_ (min g DM/mg)	K_2_ (g DM/mg)	R^2^	RMSE	R_0_ (mg/g DM min)	Ye (mg/g DM)
Untreated	0.124 ± 0.032	0.180 ± 0.000	0.998	0.082	8.638 ± 2.212	5.565 ± 0.014
US	0.120 ± 0.019	0.181 ± 0.005	0.996	0.139	7.784 ± 0.549	5.531 ± 0.074
SC-CO_2_	0.095 ± 0.004	0.151 ± 0.001	0.998	0.108	10.517 ± 0.489	6.636 ± 0.037
SC-CO_2_ + US	0.057 ± 0.002	0.139 ± 0.000	0.997	0.136	17.697 ± 0.472	7.194 ± 0.007

## Data Availability

All related data and methods are presented in this paper. Additional inquiries should be addressed to the corresponding author.
